# Possible Mechanical Stabilization of the Photoreceptor Connecting Cilium: From Molecular Scaffolds to Drug Repurposing

**DOI:** 10.3390/cells15211931

**Published:** 2026-10-23

**Authors:** Marianna Weener, Konstantin Kotliar

**Affiliations:** 1 Broad Institute, Cambridge, MA 02142, USA; 2 Department of Medical Engineering and Technomathematics, Aachen University of Applied Sciences, Campus Juelich, 52428 Juelich, Germany; kotliar@fh-aachen.de

**Keywords:** photoreceptor, connecting cilium, ciliopathy, mechanobiology, drug repurposing, retinitis pigmentosa, *FAM161A*, *CEP290*, biomechanics, neuroprotection

## Abstract

**Highlights:**

**What are the main findings?**

- The photoreceptor connecting cilium contains a FAM161A–POC5–centrin scaffold that maintains microtubule cohesion and is essential for photoreceptor structural integrity.
- Connecting-cilium biomechanics remain largely unexplored, while current therapies primarily target genetic, trafficking, or neurodegenerative mechanisms, not mechanical stability. Improved connecting cilium stability might help slow vision deterioration in patients with different ciliary gene mutations, offering an effective, partially gene-agnostic therapeutic strategy.

**What are the implications of the main findings?**

- Mechanical compromise of the connecting cilium represents a testable therapeutic hypothesis, but direct biomechanical measurements are needed before mechanical stabilization can be considered a treatment strategy.
- A research roadmap combining biomechanical characterization, targeted delivery, engineered biomaterials, and retinal disease models could enable future gene-agnostic approaches to photoreceptor protection.

**Abstract:**

Vertebrate rod and cone photoreceptors are among the most morphologically extreme cells in human biology, resembling very thin sticks with elongated outer segments connected to the inner segments by a narrow connecting cilium (CC) of approximately 200–300 nm in diameter. This specialized organelle serves as the sole conduit for the massive daily protein and membrane trafficking required for outer segment renewal, while simultaneously providing structural anchorage. Dysfunction of the CC and its associated molecular machinery, most directly the failure of protein and lipid trafficking across the cilium, underlies numerous retinal ciliopathies, including retinitis pigmentosa (RP) and Leber congenital amaurosis (LCA). Recent ultrastructural studies have revealed an inner protein scaffold within the CC comprising FAM161A, POC5, and centrin that acts as a “structural zipper” maintaining microtubule cohesion. Despite these advances, the mechanical properties of the CC remain uncharacterized, and whether biomechanical compromise of the CC contributes to ciliopathy progression is unknown. This review critically synthesizes current knowledge of photoreceptor CC structure, biomechanics, and dysfunction; evaluates existing and repurposed pharmacological agents that may protect photoreceptors through ciliary or non-ciliary mechanisms; and assesses the speculative but intriguing concept of exogenous organelle-scale mechanical stabilization. We identify critical gaps at the intersection of mechanobiology, nanobiomaterials, and retinal therapeutics, proposing a research agenda for testing whether biomechanical insights can be translated into clinically actionable strategies.

## 1. Introduction

**Figure 1:**
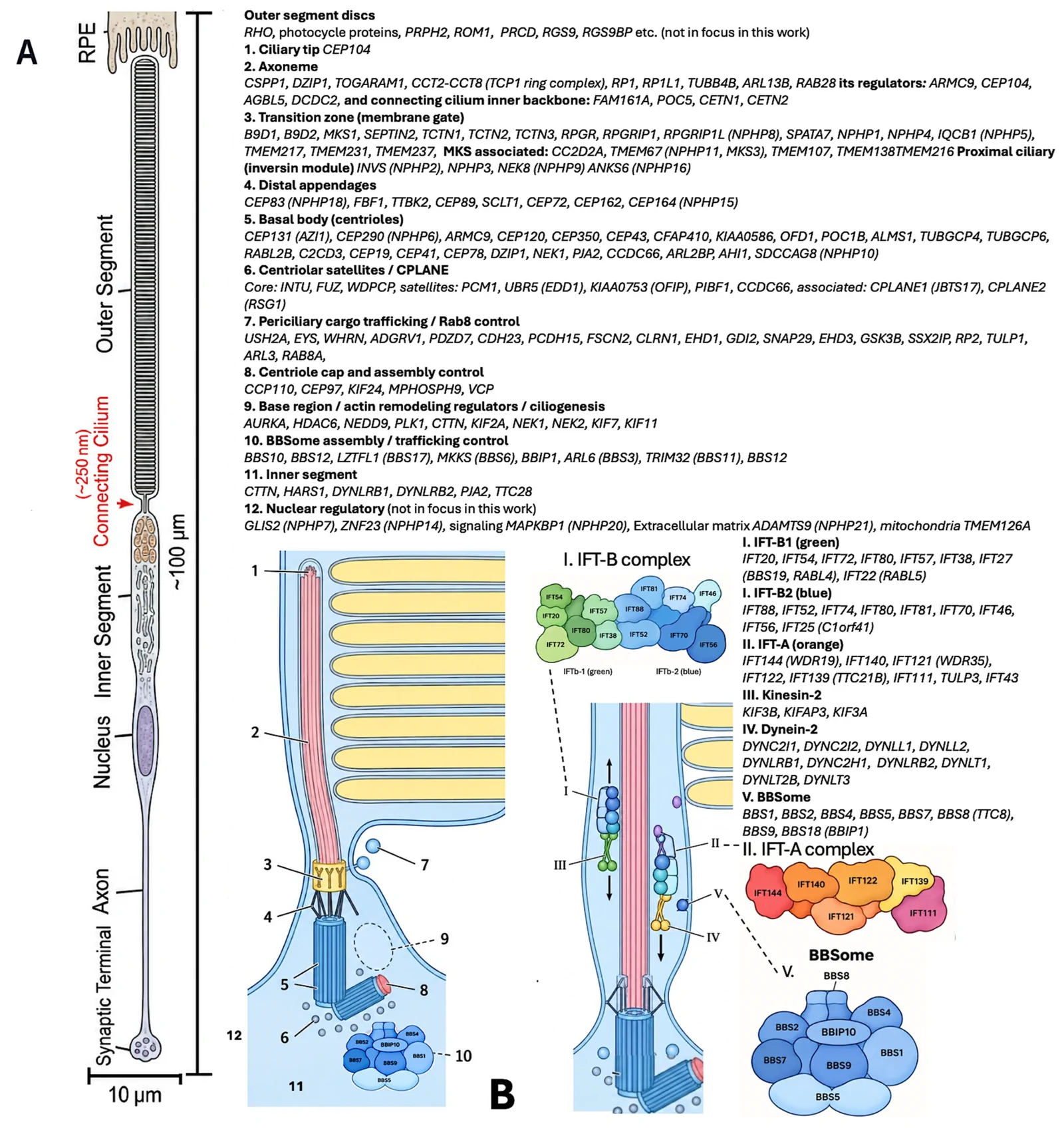
(**A**) Anatomy of the human rod photoreceptor with the accent on connecting cilium (CC). Schematic representation of a rod photoreceptor drawn to realistic scale, illustrating the extreme elongation of the cell (length-to-width ratio ~33:1). (**B**) Detailed structure of the connecting cilium, nascent disks, and intraflagellar trafficking machinery including IFT and BBSome, depicting the proteins that form its main structural and transport components, classified into groups. This figure aims to provide an approach for future stratification of gene defects that may be amenable to subcellular interventions aimed at stabilizing the CC, while identifying those for which such approaches are unlikely to be beneficial.

Rod photoreceptors possess one of the most extreme aspect ratios in human cell biology. A single rod cell measures approximately 100 μm in length but only 250–300 nm in diameter at its narrowest point, yielding an aspect ratio of roughly 30:1–50:1 depending on species and measurement criteria (Figure 1A,B) [1,2]. The outer segment (OS) is a highly organized stack of ~1000 membranous discs containing the phototransduction machinery and is connected to the inner segment (IS) by the connecting cilium (CC), a specialized non-motile cilium of approximately 0.2 μm in diameter and 1.5 μm in length [3,4]. This narrow bridge must simultaneously serve two seemingly contradictory functions: structural anchorage of the massive OS to the IS, and unimpeded bidirectional trafficking of proteins, lipids, and membrane components required for the daily renewal of approximately 7–10% of the OS [5,6]. Notably, an estimated 30–50% of retinitis pigmentosa cases are attributable to defects in the structure or function of the connecting cilium [3].

**Figure 2:**
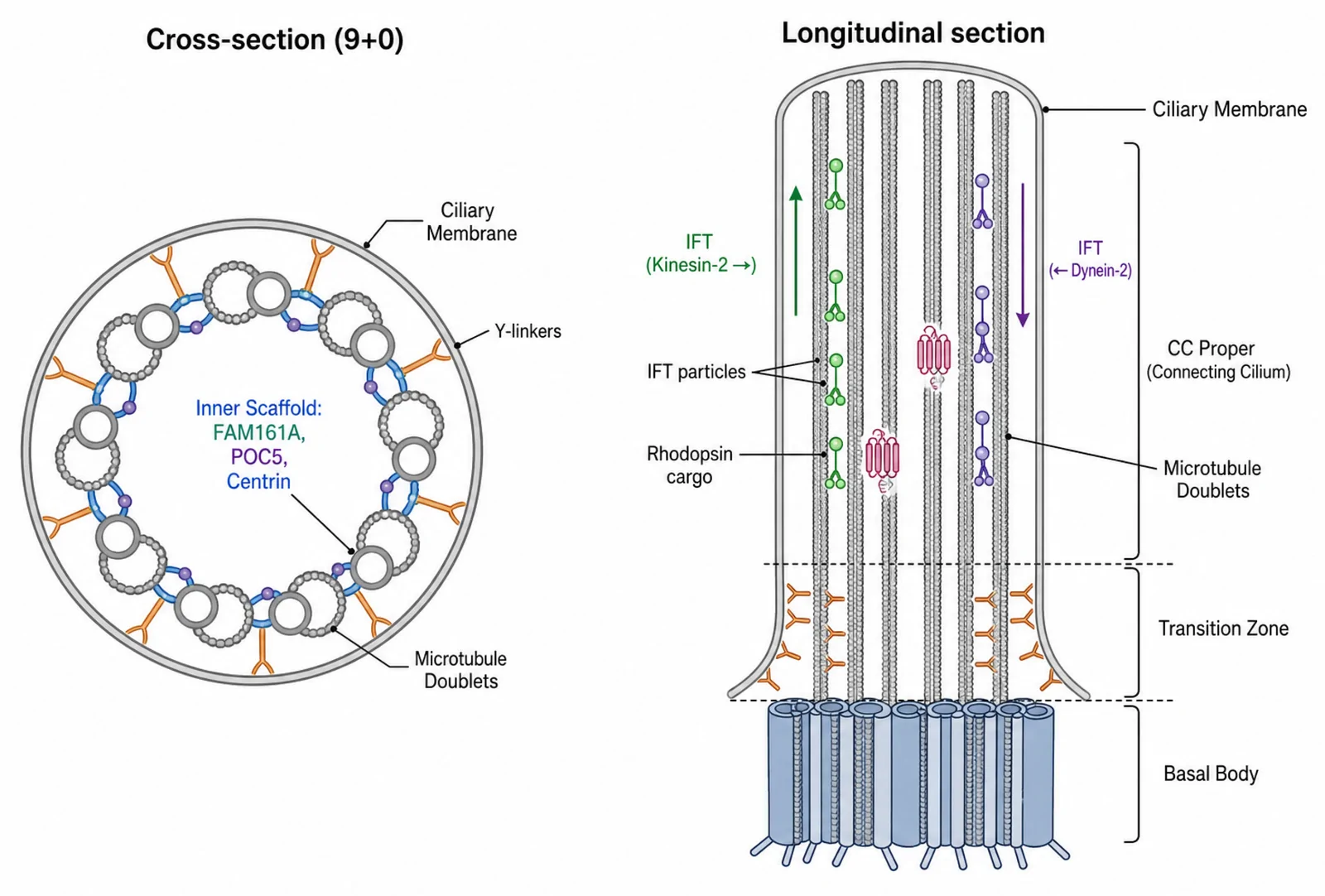
Molecular architecture of the photoreceptor connecting cilium. (**Left**) Cross-sectional view of the CC showing the 9+0 axoneme: nine microtubule doublets arranged in a ring, bridged by the inner scaffold proteins FAM161A, POC5, and centrin, which maintain axonemal cohesion. Y-shaped linkers connect the doublets to the surrounding ciliary membrane. (**Right**) Longitudinal view showing the basal body, the transition zone with Y-linkers forming the ciliary gate, and bidirectional intraflagellar transport (IFT): anterograde trafficking driven by kinesin-2 and retrograde trafficking by dynein-2, carrying cargo such as rhodopsin toward the outer segment.

The CC is not merely a passive conduit. It is a highly organized molecular assembly featuring a 9+0 microtubule doublet axoneme, Y-shaped linkers connecting microtubules to the ciliary membrane (Figure 1B and Figure 2), and a transition zone that functions as a selective diffusion barrier [4,7]. Recent cryo-electron tomography (cryo-ET) and ultrastructure expansion microscopy (U-ExM) studies have revealed an inner protein scaffold within the CC composed of FAM161A, POC5, and centrin that bridges neighboring microtubule doublets and acts as a “structural zipper” to maintain axonemal cohesion [8,9]. Loss of these scaffold proteins leads to microtubule spreading, CC disorganization, and progressive photoreceptor degeneration, demonstrating that CC structural integrity is required for photoreceptor viability [8,10,11].

Despite the critical importance of CC structural integrity, therapeutic research for retinal ciliopathies has focused almost exclusively on genetic correction (gene therapy, CRISPR editing) and molecular pharmacology (neuroprotective small molecules, neurotrophic factors) [12,13,14]. Biomechanical aspects of CC dysfunction and the possibility of its exogenous stabilization have received virtually no attention in the published literature. This gap is notable for several reasons: the CC is continuously exposed to mechanical loads arising from OS renewal, ocular micromovements, and intraocular pressure dynamics; many ciliopathy mutations directly compromise structural proteins; and the field of mechanobiology has matured sufficiently to offer tools for organelle-scale mechanical measurement and intervention [15,16]. At the same time, it must be stated clearly that impaired trafficking of proteins and lipids across the CC is the established driver of photoreceptor degeneration in ciliopathies.

The potential contribution of biomechanical aspects of the CC to disease pathology remains an open, testable question rather than an established mechanism, and is treated as such throughout this review.

This review critically examines the current state of knowledge regarding photoreceptor CC structure, biomechanics, and dysfunction. We evaluate the landscape of existing and repurposed pharmacological agents spanning small molecules, peptides, monoclonal antibodies, and other biologics that may protect photoreceptors through ciliary or non-ciliary mechanisms. We also assess the speculative concept of exogenous organelle-scale mechanical stabilization (the “bioglue” or “molecular exoskeleton” hypothesis), analyzing its scientific plausibility, potential technical feasibility, and translational challenges. Our goal is to provide a balanced, evidence-based overview of where potential mechanical stabilization of the CC might fit within the broader therapeutic landscape for retinal degeneration, and to delineate the experimental evidence that would be required to test this concept.

## 2. Photoreceptor Connecting Cilium: Structure and Molecular Architecture

### 2.1. Gross Morphology and Dimensions

The photoreceptor CC is surrounded by a specialized periciliary membrane that is continuous with both the IS plasma membrane and the OS disc membranes. The transition zone, located at the proximal end of the CC, contains Y-shaped linkers that connect microtubule doublets to the ciliary membrane and form a diffusion barrier regulating protein entry into the OS [4,7,17] (Figure 1B).

### 2.2. The Inner Protein Scaffold: FAM161A, POC5, and Centrin

A landmark 2022 study by Mercey et al. used cryo-ET and subtomogram averaging to reveal an inner scaffold within the CC that bridges neighboring microtubule doublets [8]. This scaffold is composed of three key proteins: (1) FAM161A, a microtubule-associated protein; mutations in *FAM161A* gene cause autosomal recessive RP [10,11,18]. FAM161A localizes to the CC and interacts with other ciliopathy-associated proteins, including CEP290 and RPGR [11,19]. (2) POC5, a centrin-binding protein that localizes to the distal CC and is essential for CC assembly and stability; biallelic mutations in *POC5* gene cause autosomal recessive RP [20]. (3) Centrin (coded by *CETN2* gene), a calcium-binding protein that localizes to the CC lumen and forms part of the inner scaffold [8,9].

Together, these proteins form a “structural zipper” that maintains the cohesion of microtubule doublets within the CC. Loss of FAM161A in mice leads to microtubule spreading, CC disorganization, and progressive photoreceptor degeneration, demonstrating that the inner scaffold is essential for CC structural integrity [8,10]. A 2024 study using U-ExM in canine photoreceptors confirmed the conservation of this scaffold architecture across species and revealed distinct rod–cone differences in photoreceptor sensory cilium organization, with cones exhibiting longer basal bodies and daughter centrioles, a shorter connecting cilium, and a longer and wider bulge region compared with rods [9].

### 2.3. Transition Zone Proteins and the Ciliary Gate

The transition zone (TZ) at the distal CC contains a complex protein network that regulates ciliary trafficking. Key TZ proteins include: (1) CEP290 (NPHP6), a large coiled-coil protein that anchors the TZ to the microtubule cytoskeleton; mutations in *CEP290* gene cause LCA, Senior–Løken syndrome, and Joubert syndrome [21,22]. CEP290 protein is essential for maintaining OS protein polarity and CC stability [23]. (2) RPGR (retinitis pigmentosa GTPase regulator), a guanine nucleotide exchange factor for RAB8A that regulates cargo delivery to the OS; *RPGR* gene mutations cause X-linked RP [24,25]. (3) RPGRIP1, an RPGR-interacting protein 1 that anchors RPGR to the TZ; *RPGRIP1* gene mutations cause LCA and cone–rod dystrophy [26,27]. (4) NPHP proteins (NPHP1, NPHP4, and other family members), components of the NPHP complex at the TZ that regulate ciliary protein composition [28].

### 2.4. Intraflagellar Transport (IFT) and Ciliary Trafficking

The CC is the sole conduit for all OS-destined proteins. Intraflagellar transport is mediated by kinesin-2 (anterograde) and dynein-2 (retrograde) motors, which move cargo along the CC axoneme [29,30]. Key IFT protein components in photoreceptors include IFT20, IFT88, IFT140, and the BBSome complex [29,31,32]. Rhodopsin, the most abundant OS protein (~10^7^ molecules per rod), is transported through the CC via IFT-dependent mechanisms [33,34]. The sheer volume of daily trafficking is estimated at ~2000 rhodopsin molecules per minute per rod and places extraordinary demands on CC transport capacity [5,35].

## 3. Ciliopathies and Retinal Degeneration

### 3.1. Genetic Basis of Retinal Ciliopathies

Retinal ciliopathies encompass a spectrum of inherited disorders caused by mutations in genes encoding ciliary proteins, including the scaffold and transition-zone proteins described in Section 2. The most common include: retinitis pigmentosa (RP), a genetically heterogeneous group of disorders affecting ~1 in 4000 individuals, with over 100 implicated genes, many of which encode ciliary proteins (*FAM161A*, *RPGR*, *RPGRIP1*, *CEP290*, *POC5*, and others) [12,36]; Leber congenital amaurosis (LCA), a severe early-onset retinal dystrophy most commonly caused by *CEP290* mutations, which account for ~15–20% of cases [21,37]; Bardet–Biedl syndrome (BBS), a multisystem ciliopathy caused by mutations in BBSome components and featuring retinal degeneration, obesity, polydactyly, and renal dysfunction [38]; and Senior–Løken syndrome, characterized by retinal degeneration and nephronophthisis and caused by mutations in *NPHP* genes [28].

### 3.2. Mechanisms of Photoreceptor Degeneration in Ciliopathies

Photoreceptor degeneration in ciliopathies arises through multiple, often overlapping mechanisms: (1) structural disorganization –loss of CC scaffold proteins (FAM161A, POC5) leads to microtubule disorganization and OS collapse [8,10]; (2) trafficking defects –mutations in IFT components, cargo adaptors, or TZ proteins impair protein delivery to the OS, leading to rhodopsin mislocalization and OS shortening [29,31,39]; and (3) proteostasis imbalance –accumulation of misfolded proteins (e.g., mutant rhodopsin in P23H *RHO* RP) triggers endoplasmic reticulum (ER) stress and activation of the unfolded protein response (UPR) [40,41]. In addition, recent evidence suggests that premature ciliary disassembly may be a pathogenic event in some forms of retinal degeneration [42]. Notably, the first two mechanisms converge on a common endpoint: failure to transport proteins and lipids across the CC, which is the established proximal cause of photoreceptor degeneration in these disorders.

### 3.3. Altered Connecting Cilium Biomechanics

While the genetic and biochemical mechanisms of ciliopathies are well documented, the biomechanical consequences of CC dysfunction have received little attention. Under physiological conditions, the CC must withstand axial tension during OS renewal and disc shedding, shear forces from daily disc turnover (~7–10% of OS mass per day), bending moments from ocular movements and saccades, and cyclic compressive stress from intraocular pressure dynamics. Importantly, as in any living tissue, the molecular components of the CC turn over continuously; healthy CCs therefore do not appear to accumulate mechanical damage over a lifetime, and classical material-fatigue models cannot be applied to the cilium literally.

Undoubtedly, the failure to transport proteins and lipids across the CC is the primary event leading to photoreceptor degeneration. However, mutations that compromise CC structural proteins might reduce the organelle’s resistance to these physiological mechanical stresses, presumably accelerating degeneration. It can also be assumed that, in at least some cases, an interplay of biomechanical compromise and impaired transport function underlies CC dysfunction. We stress that this biomechanical-contribution hypothesis remains untested: quantitative biomechanical measurements of the CC, i.e., bending stiffness, tensile strength, and resistance to cyclic loading, are lacking in the published literature, and no study has directly demonstrated that mechanical stress on the photoreceptor CC contributes to the pathology of any retinal disease. This represents an undervalued gap in our understanding of ciliopathy pathogenesis.

## 4. Biomechanics of the Connecting Cilium

### 4.1. Biomechanical Properties of Primary Cilia Outside the Retina

While the biomechanical properties of the photoreceptor CC have not been directly measured, studies of primary cilia in other cell types provide relevant context. Schwartz et al. (1997) measured the primary cilium response to fluid shear stress using fluid shear bending assays; the flexural rigidity of kidney primary cilia was estimated at 1.4–3.1 × 10^−23^ N·m^2^ [43]. More recent measurements using micro-tensile testing and atomic force microscopy (AFM) have yielded Young’s moduli in the range of 0.1–1 MPa for isolated primary cilia [44]. Recalculating the Young’s modulus from Schwartz’s data, assuming a cilium diameter of 205 nm, yields a value in the same range (~0.35 MPa). Mathematical models of cilium bending under fluid flow have confirmed that ciliary mechanics are governed by basal anchorage stiffness and anisotropic microtubule moduli [45,46].

Importantly, there is experimental evidence that pathological conditions change the mechanical stiffness of cilia. Resnick used optical trapping to bend individual primary cilia of kidney epithelial MDCK (Madin–Darby Canine Kidney) cells and found that stabilization of hypoxia-inducible factor (HIF), which is a hallmark of hypoxic and von Hippel–Lindau-deficient states, softens the cilia, reducing their flexural rigidity almost twofold [47]. Kumamoto et al. studied primary cilia from PCK (polycystic kidney) rats carrying a *Pkhd1* gene mutation and described the cystic cilia as having decreased stiffness. Their mathematical model predicts drastically altered force transmission, with the long, curved, diseased cilium experiencing only about one quarter of the drag force of the short, straight configuration under equivalent flow conditions [48]. Although these data come from renal rather than retinal cilia, they demonstrate that ciliopathy-associated molecular defects can measurably alter ciliary mechanics, which is a prerequisite for any hypothesis in which altered CC biomechanics contributes to retinal disease.

### 4.2. Extrapolation to the Photoreceptor Connecting Cilium

The photoreceptor CC differs from canonical primary cilia in several important respects. Its diameter (~250–300 nm) is comparable to that of primary cilia (~250 nm), but its 9+0 axoneme lacks the central microtubule pair found in motile 9+2 cilia, which reduces stiffness. On the other hand, its length (1–1.5 μm) is shorter than that of many primary cilia (~2–10 μm), which may increase its bending stiffness. Functionally, the CC is also unusual among cilia: rather than serving a primarily sensory role, it bears a substantial structural load, anchoring the entire OS (~20–30 μm in rods) to the IS. In addition, the CC must accommodate continuous bidirectional IFT, which may impose further mechanical constraints. No published study has measured the biomechanical properties of the photoreceptor CC directly, neither under physiological nor under pathological conditions. This gap needs to be addressed, as the CC’s biomechanical behavior is entirely unknown; insights into this question may shed light on future treatment development.

### 4.3. Biomechanical Failure Modes

Based on the CC’s structure and function, several biomechanical failure modes can be hypothesized:

(1) Microtubule doublet spreading –loss of inner scaffold cohesion leads to axonemal disorganization (e.g., *FAM161A* deficiency) [8];

(2) Membrane rupture –excessive bending or shear could compromise the periciliary membrane, disrupting the diffusion barrier;

(3) Buckling or excessive deformation under cyclic physiological loading –although axonemal components turn over continuously, a cilium assembled from compromised structural proteins may deform beyond the range compatible with normal function;

(4) IFT obstruction –mechanical deformation of the CC could impede IFT, creating a vicious cycle of trafficking failure and OS degeneration.

These failure modes are speculative but testable. Finite-element modeling, informed by cryo-ET structural data, might potentially predict CC biomechanical behavior under various loading conditions and identify critical weak points.

## 5. Current Therapeutic Strategies for Retinal Ciliopathies

### 5.1. Gene Therapy

Gene augmentation therapy has achieved landmark success in *RPE65*-associated LCA, with voretigene neparvovec (Luxturna) receiving FDA approval in 2017 [49,50]. For ciliopathy genes, several approaches are in clinical development: EDIT-101, a CRISPR–Cas9-based therapy targeting the common IVS26 mutation in *CEP290* gene, showed promising results in a phase 1/2 trial, with 64% of participants showing meaningful improvements in vision-related outcomes [51]; multiple AAV-based gene therapy trials are ongoing for X-linked RP caused by *RPGR* mutations [52]; and *FAM161A* gene augmentation is being studied preclinically [53]. Gene therapy offers the potential for definitive correction of the underlying genetic defect but faces challenges including AAV cargo capacity (*CEP290* is ~7.5 kb, exceeding AAV capacity), immune responses, and the need for early intervention before irreversible photoreceptor loss [13,54].

### 5.2. Neurotrophic Factors

Ciliary neurotrophic factor (CNTF) is a 22 kDa cytokine that has been extensively studied as a neuroprotective agent for photoreceptors. Encapsulated cell-based delivery of CNTF (NT-501, Neurotech Pharmaceuticals, Cumberland, RI, USA 02864) showed safety in phase I trials [55], but a randomized controlled trial failed to demonstrate significant functional benefit in RP patients [56]. CNTF acts primarily through Müller glial cells via gp130 receptor activation, inducing downstream cytokine release [57]. While its clinical efficacy for photoreceptor protection has been disappointing [58], CNTF remains under investigation for other retinal indications, including macular telangiectasia type 2.

Glial cell line-derived neurotrophic factor (GDNF) has protected photoreceptors in some models, but delivery challenges limit clinical translation [59]. Brain-derived neurotrophic factor (BDNF) has shown neuroprotective effects in retinal ganglion cell models, but effects on photoreceptors are less established [60]. Oncostatin M (OSM) is a gp130-family cytokine that, like CNTF, acts through Müller glia; nanoparticle delivery of OSM showed photoreceptor protection in rodent models [61], albeit at an early stage of research.

### 5.3. Small-Molecule Neuroprotectants

Several small molecules have shown promise in preclinical models of retinal degeneration. Reserpine, identified in a high-content screen, maintains photoreceptor survival in ciliopathy models by resolving proteostasis imbalance and ciliogenesis defects [40]. A 2025 study showed sex-specific attenuation of photoreceptor degeneration in the P23H *RHO* rat model [62]. However, reserpine’s known side effects (depression, hypotension, parkinsonism) limit its chronic use, and its mechanism of photoreceptor protection is not fully understood. N-acetylcysteine (NAC) is an antioxidant that promotes cone survival in RP models [63]. A phase I clinical trial showed that oral NAC was safe and produced modest improvements in cone function in RP patients [64]. NAC is particularly attractive for repurposing given its excellent safety profile, oral bioavailability, and low cost; however, its effects are modest and may be limited to cone preservation rather than rod protection.

Valproic acid is an anticonvulsant, mood stabilizer, and histone deacetylase inhibitor with mixed results: protective in *rd1* mice but potentially harmful in *rd10* mice [65]. A small pilot study in RP patients showed transient improvement in visual acuity and visual field during treatment [66], but larger trials are needed. The conflicting preclinical results and potential for serious side effects (hepatotoxicity, teratogenicity) warrant caution [67].

Rapamycin (sirolimus) is an immunosuppressant and mTOR inhibitor that activates autophagy and protects photoreceptors in *rd1* mice and retinal detachment models [68,69]. However, rapamycin showed differential effects on rods (protective) and cones (potentially harmful) in light-stress models [70], highlighting the complexity of mTOR signaling in photoreceptor biology. Curcumin has anti-inflammatory and antioxidant properties but limited ocular bioavailability [71]. Ursodeoxycholic acid (UDCA) is a bile acid with anti-apoptotic properties that protected photoreceptors in some models [72]. Sildenafil is a *PDE5* inhibitor; paradoxically, *PDE6* inhibitors can cause retinal toxicity, but low-dose *PDE5* inhibition may have neuroprotective effects through cGMP modulation [73].

### 5.4. Complement Inhibitors

The complement cascade has emerged as a therapeutic target in geographic atrophy (GA) secondary to AMD. Pegcetacoplan (C3 inhibitor) and avacincaptad pegol (C5 inhibitor) both reduce GA lesion growth by ~20–27% over 12 months [74,75]. Importantly, pegcetacoplan was associated with reduced photoreceptor degeneration beyond areas of GA [76], suggesting a broader neuroprotective effect. However, neither agent has demonstrated functional improvement in visual acuity, raising questions about clinical relevance [77].

## 6. Off-Label Drug Repurposing for Photoreceptor Protection

### 6.1. Rationale for Drug Repurposing

**Figure 3:**
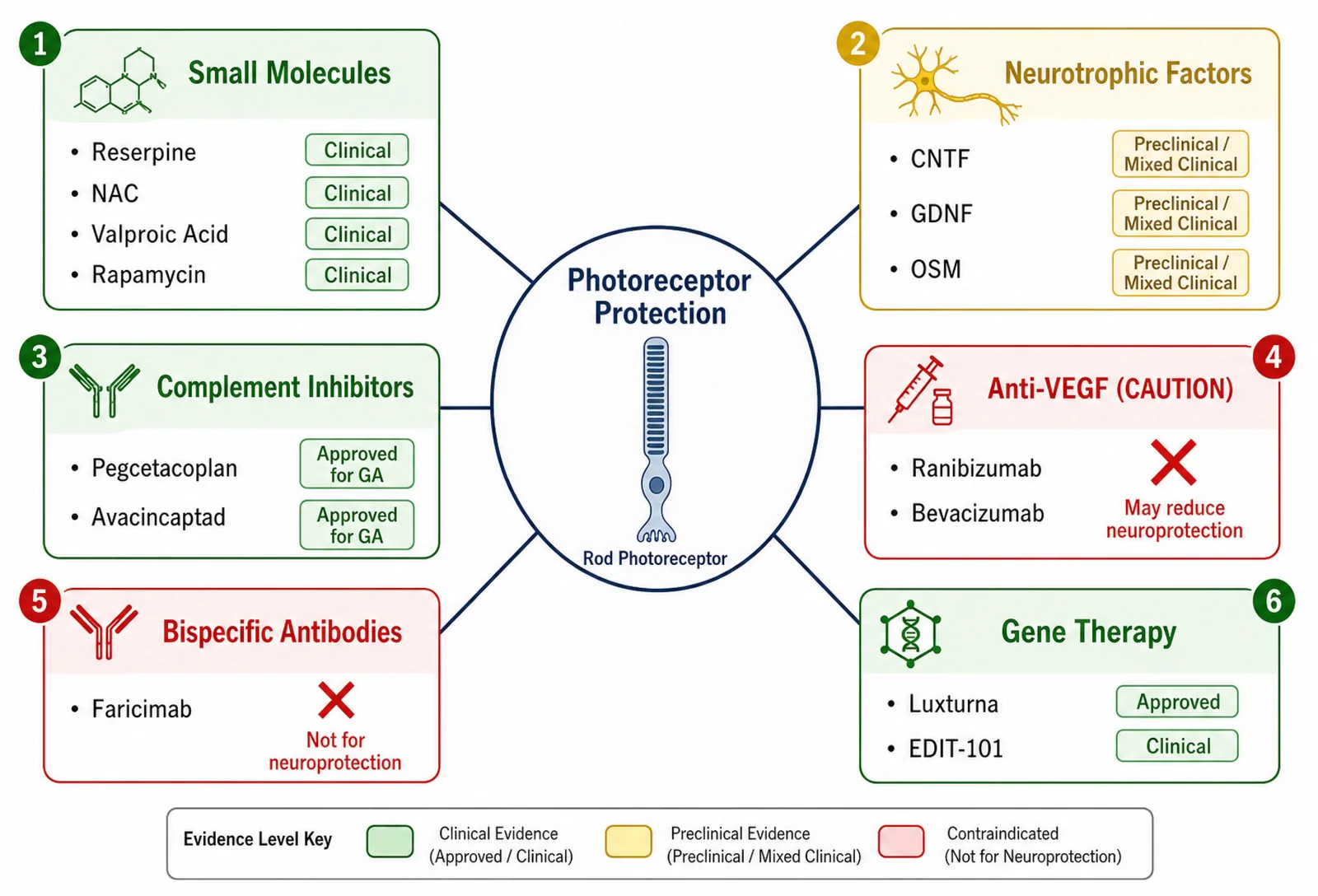
Landscape of treatment candidates for photoreceptor protection in connecting cilium damaging conditions. Overview of six therapeutic classes evaluated in this review, color-coded by evidence level: green, clinical evidence or approved therapy; yellow, preclinical or mixed clinical evidence; red, contraindicated for photoreceptor protection. Small molecules (reserpine, N-acetylcysteine, valproic acid, rapamycin) and neurotrophic factors (CNTF, GDNF, oncostatin M) act through proteostasis, antioxidant, autophagy, or gliotrophic mechanisms. Complement inhibitors (pegcetacoplan, avacincaptad pegol) are approved for geographic atrophy and reduce photoreceptor degeneration beyond lesion borders. Anti-VEGF agents (ranibizumab, bevacizumab) and the bispecific antibody faricimab are contraindicated in this context because VEGF-A is an endogenous retinal neuroprotective factor. Ongoing gene therapy trials (for *RPGR*, *LCA5* and planned for *PRCD* and *PCARE*; approved drug for *RPE65 *–Luxturna –is indirect, not primarily cilium defect) or gene editing approaches (EDIT-101, CRISPR-Cas9 for *CEP290*) address the underlying genetic defects and provide gene-specific treatment of tiny fraction of patients with connecting cilium defects.

Identification of new therapeutic indications for existing approved drugs offers several advantages for retinal ciliopathies: established safety profiles, known pharmacokinetics, reduced development costs, and faster translation to clinical trials [78,79]. For rare diseases like RP, where traditional drug development is economically challenging, repurposing is particularly attractive. Candidates relevant to treatment of connecting cilium disease are summarized in Table 1 and Figure 3.

### 6.2. Theoretical Biologics for CC Stabilization

No existing biologics directly target CC structural proteins. Several theoretical approaches could nevertheless be considered: (1) anti-inflammatory mAbs targeting TNF-α, IL-6, or other cytokines that contribute to photoreceptor degeneration in the setting of ciliopathy-associated inflammation; (2) neurotrophic factor fusion proteins –engineered biologics combining neurotrophic activity with extended ocular half-life; and (3) antibody–drug conjugates targeting specific retinal cell types for the delivery of neuroprotective payloads. None of these approaches would stabilize the CC mechanically; they are included here because biologics are often overlooked in repurposing discussions for retinal diseases. All remain speculative and would require extensive preclinical validation before any clinical application.

## 7. Organelle-Scale Mechanical Stabilization

**Figure 4:**
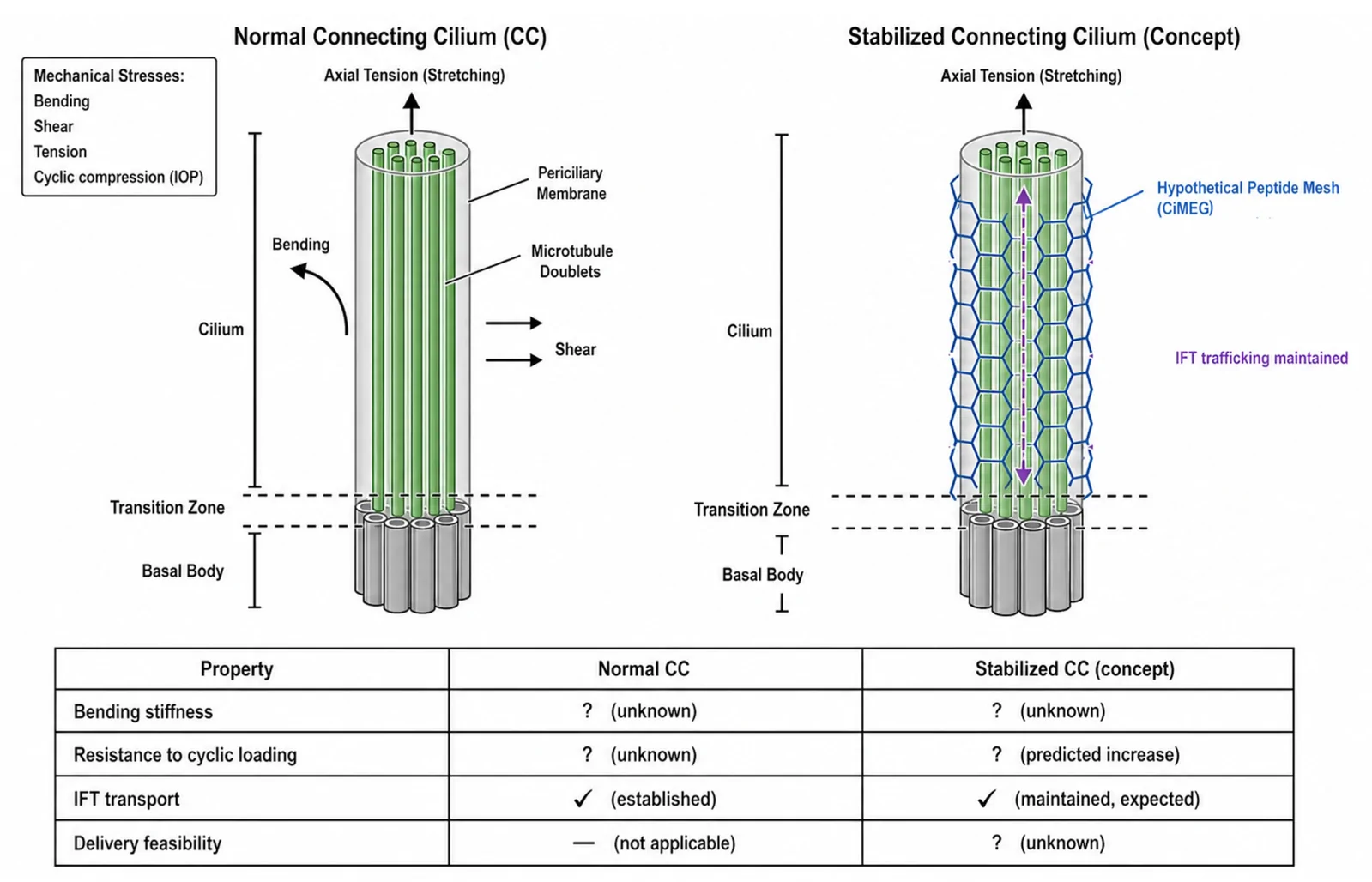
Proposed concept of exogenous mechanical stabilization of the connecting cilium (CiMEG). (**Left**) The native CC is exposed to continuous mechanical stresses, including axial tension during outer segment renewal, shear from disc shedding, bending from ocular micromovements, and cyclic compression from intraocular pressure (IOP) dynamics. (**Right**) Hypothetical stabilized CC: a porous, elastic cilium peptide mesh exogenous (CiMEG concept) assembled around the periciliary membrane is proposed to provide mechanical stabilization of affected photoreceptor, resembling biomechanical status of the native CC and increasing resilience while preserving IFT trafficking. The table summarizes predicted versus unknown properties of the concept. Checkmarks indicate properties supported by established evidence or by analogy to existing biomaterials, and question marks indicate properties that remain experimentally untested.

The idea of exogenous mechanical stabilization of the CC, variously termed a “molecular exoskeleton” or “bioglue”, proposes that a biocompatible material could be delivered to the periciliary region to reinforce the CC against mechanical stress (Figure 4). This concept, while novel, raises fundamental questions about feasibility, specificity, and safety.

Several lines of evidence suggest that the concept is not implausible. The CC possesses an endogenous inner scaffold or backbone (FAM161A/POC5/centrin) that provides mechanical cohesion [8], demonstrating that molecular-scale mechanical reinforcement is biologically relevant. Self-assembling peptides and elastin-like polypeptides (ELPs) can form viscoelastic networks at the nanoscale [80,81]. The periciliary membrane is accessible, at least in principle, from the extracellular (subretinal) space. Finally, mechanobiology tools (AFM, optical tweezers, cryo-ET) now exist to measure and manipulate organelle mechanics [44,82].

However, substantial concerns remain regarding delivery, IFT interference, and immunogenicity. The CC is only ~200–300 nm in diameter; any exogenous material must be precisely targeted to this nanoscale structure without affecting surrounding tissue. The CC is located deep within the subretinal space, and intravitreal delivery would require the agent to cross the inner limiting membrane, migrate through the retina, and localize to the CC, a formidable pharmacokinetic challenge [83,84]. The CC is the sole conduit for all OS proteins; any material that obstructs IFT, even partially, would be catastrophic for photoreceptor survival [29,33]. No known targeting mechanism exists to direct an exogenous material specifically to the CC, and the periciliary membrane shares lipid composition with other retinal membranes. Finally, repeated intraocular administration of any exogenous material risks inflammatory responses, which are particularly dangerous in the immune-privileged but inflammation-sensitive eye [85]. However, the eye is a relatively isolated organ, and overcoming the challenges described above could potentially enable the development of a molecular, self-assembling “cocktail” that envelops the CC, stabilizes its structure, and ultimately provides a gene-agnostic therapeutic approach for patients with ciliopathies leading to rod degeneration.

As an illustrative concept, the authors of this review have explored a short peptide, termed CiMEG (Figure 4), designed to mechanically stabilize the photoreceptor CC in a gene-agnostic manner when CC instability has a genetic cause. CiMEG comprises four domains, each with a specific molecular function: (1) a membrane-anchoring amphipathic helix (KRLALALKKALALAA) even though in vivo, this peptide risks disrupting membrane integrity and lysing the periciliary membrane, so additional modification might be needed; (2) an elastin-like self-assembly core (GPGVPGVGPGVPG) based on the well-characterized VPGXG motif, although reliable self-assembly generally requires ELP sequences longer than 100 amino acids, shorter isoforms might remain an unstructured random coil in aqueous solution and might not form a supramolecular viscoelastic mesh; (3) a weak, promiscuous protein-interaction motif (EDYF) for association with CC proteins, even though the subretinal space is dense with the interphotoreceptor matrix, rich in glycosaminoglycans and chondroitin sulfate proteoglycans, an amphipathic, cationic peptide with promiscuous motifs might non-specifically aggregate within the matrix before reaching the CC, so targeting will be required; and (4) a protease-sensitive clearance tail, although the subretinal space has limited proteolytic activity, and peptide clearance from the eye occurs primarily through diffusion and bulk flow [83].

If successful, such an externally synthesized peptide therapy would extend the current therapeutic paradigm –DNA-, RNA-, and protein-level medicines –toward direct subcellular structural intervention, in effect a form of “subcellular surgery”. The limitations and challenges of such an externally synthesized CC-stabilizing sleeve include the following: membrane anchoring must be specific enough to engage only the CC region; the elastin-like polypeptide must be long enough to ensure reliable self-assembly; and methods must be developed to measure CC bending stiffness before and after application of the sleeve in order to prove the concept. The goal should not be to maximize stiffness of the CC. It should be to restore normal biomechanical integrity and geometry of the photoreceptor. Also, outer segment renewal requires constant evagination of the ciliary plasma membrane at the distal ciliary tip. An exogenous crosslinked sleeve surrounding the periciliary membrane risks freezing membrane fluidity and blocking vesicular transport/membrane evagination, which would directly accelerate retinal degeneration.

A further critical consideration is the route and specificity of administration. Any scaffold intended to stabilize the CC would have to be delivered locally by subretinal, intravitreal, or suprachoroidal injection with a proper targeting ligand to reduce any unwanted off-target systemic effect. Cilia are ubiquitous organelles: motile cilia line the bronchi and the epididymis, and primary cilia are present in kidney tubules and most other tissues. Systemic administration of a cilia-stabilizing agent could therefore alter the mechanics of cilia throughout the body with unknown and potentially harmful consequences. This is particularly concerning for motile cilia, whose function depends on precisely regulated beating and whose stiffening could impair mucus clearance or sperm transport. Local ocular delivery is thus not merely a preference but a safety requirement. The eye is, in this respect, a favorable target organ: it is small, compartmentalized, and surgically accessible, and intraocular injection is routine in clinical practice. Nevertheless, even local delivery would need to achieve CC-specific targeting within the retina to avoid mechanically perturbing other ciliated cell types in and around the eye.

No published study has attempted exogenous mechanical stabilization of the CC. However, the broader concept of organelle-scale mechanical intervention has precedents. Cytoskeletal stabilizers such as paclitaxel (Taxol), which strengthen microtubules and are used clinically as a chemotherapeutics, demonstrate that small molecules can modulate cytoskeletal mechanics, even though they are not targeted at cilia [86]. Similarly, extracellular matrix mimetics such as hydrogels and nanofibers provide mechanical support to cells and tissues, though at much larger scales [87,88]. Cell-penetrating peptides and nanoparticles have been used to deliver functional materials into cells, though not specifically for mechanical stabilization [89].

Even if the scientific challenges could be overcome, the translational pathway for a CC-stabilizing agent faces significant obstacles that are not only biological but also regulatory:

− A mechanical stabilizer could be classified as a device or combination product, adding regulatory complexity;
− Peptide synthesis at pharmaceutical scale is well established, but ensuring consistent self-assembly and mechanical properties across batches would be challenging;
− Demonstrating efficacy would require novel clinical endpoints (e.g., in vivo CC biomechanical measurements), which do not currently exist.

**Figure 5:**
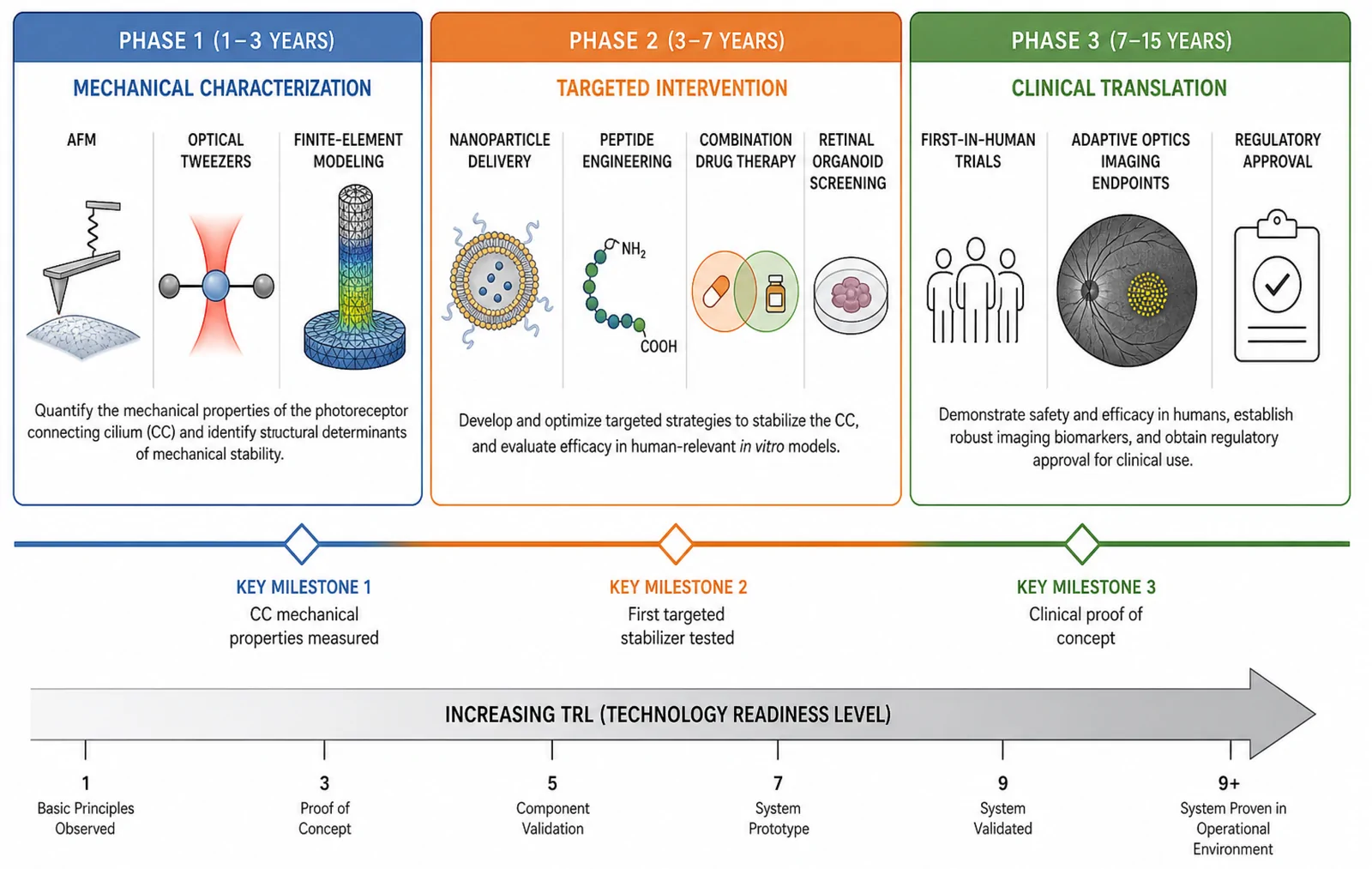
Research roadmap toward mechanical stabilization of the connecting cilium. Three-phase translational agenda mapped against Technology Readiness Level (TRL). Phase 1 (1–3 years): biomechanical characterization of the intact and damaged CC using atomic force microscopy, optical tweezers, and finite-element modeling informed by cryo-electron tomography. Phase 2 (3–7 years): targeted intervention, including CC-directed delivery systems, engineered peptide scaffolds, combination drug therapy, and mechanical stress paradigms in retinal organoids. Phase 3 (7–15 years): clinical translation, with first-in-human trials, adaptive optics imaging endpoints, and regulatory approval. Diamonds mark key milestones: measurement of CC biomechanical properties, first targeted stabilizer tested, and clinical proof of concept.

Figure 5 summarizes a proposed three-phase research roadmap toward mechanical stabilization of the CC, mapped against Technology Readiness Levels (TRLs). In Phase 1 (approximately 1–3 years), the priority is biomechanical characterization of the CC itself, measuring bending stiffness, tensile strength, and resistance to cyclic loading using AFM and optical tweezers, complemented by finite-element modeling informed by cryo-ET structural data. Without such baseline measurements, any stabilization attempt would be blind. Additionally, this phase should involve obtaining information and carrying out measurements and biomechanical modelling of photoreceptors with damaged CC. The quantitative changes in biomechanical properties in pathology should be demonstrated. In Phase 2 (approximately 3–7 years), targeted interventions could be developed and tested: CC-directed delivery systems, engineered peptide scaffolds such as the CiMEG concept outlined above, combination drug therapy, and mechanical stress paradigms in retinal organoids as disease models. In Phase 3 (approximately 7–15 years), successful candidates would move toward clinical translation, including first-in-human trials with adaptive optics imaging endpoints and, ultimately, regulatory approval. We present this roadmap not as a prediction but as a structured agenda: each phase defines the evidence that must exist before the next phase can responsibly begin.

## 8. Conclusions

The photoreceptor connecting cilium is a remarkable organelle that must balance two seemingly contradictory functions: structural anchorage of the massive outer segment and unimpeded bidirectional trafficking of proteins and membranes. Recent ultrastructural studies have revealed an inner scaffold composed of FAM161A, POC5, and centrin proteins that acts as a molecular “zipper” maintaining CC cohesion; loss of this scaffold leads to photoreceptor degeneration, demonstrating that CC structural integrity is essential for retinal health.

Despite these advances, the biomechanical properties of the CC remain entirely uncharacterized, and no therapeutic strategy directly targets CC mechanical stability. It is important to state clearly that impaired trafficking across the CC and not mechanical failure is the established cause of photoreceptor degeneration in ciliopathies; whether mechanical compromise of the CC contributes to disease progression is a hypothesis that awaits direct experimental testing. Current approaches, such as gene therapy, neurotrophic factors, and small-molecule neuroprotectants address genetic and biochemical defects but not altered CC biomechanics. Drug repurposing offers a pragmatic path to near-term photoreceptor protection, with agents such as NAC, reserpine, and rapamycin showing promise in preclinical models and early clinical trials. However, none of these agents directly stabilize the CC.

The concept of exogenous organelle-scale mechanical stabilization, a “molecular exoskeleton” for the CC is scientifically intriguing but faces formidable challenges in delivery, specificity, IFT compatibility, and regulatory translation. Any such agent would have to be administered locally to avoid affecting cilia elsewhere in the body. The critical first step is biomechanical characterization of the CC itself. Without knowing the biomechanical properties of the intact and damaged CC and the entire photoreceptor, and the forces that the CC must withstand, any attempt at mechanical stabilization would be futile. We therefore propose that the intersection of mechanobiology, cell biomechanics, nanobiomaterials, and retinal therapeutics represents a fertile frontier for innovation. By combining biomechanical characterization, computational modeling, and targeted drug delivery, it may become possible to develop first-in-class, gene-agnostic therapies that protect photoreceptors by stabilizing their most vulnerable structural element, complementing existing genetic and pharmacological approaches and offering a new paradigm for treating retinal degeneration.

## Figures and Tables

**Table 1:** Treatment candidates for photoreceptor protection in connecting cilium diseases.

Agent	Class	Mechanism	Evidence Level	CC-Specific	Feasibility
Reserpine	Small molecule	Proteostasis, ciliogenesis	Clinical	Indirect	Moderate (side effects)
NAC	Small molecule	Antioxidant	Phase I clinical	No	High (safe, cheap)
Valproic acid	Small molecule	HDAC inhibition	Clinical	No	Low (safety concerns)
Rapamycin	Small molecule	mTOR inhibition, autophagy	Clinical	No	Moderate (rod/cone selectivity)
CNTF	Peptide	Neurotrophic (Müller glia)	Preclinical	No	Moderate (delivery)
Pegcetacoplan	Peptide (PEGylated)	Complement C3 inhibition	Phase III clinical (GA)	No	High (approved for GA)
Anti-VEGF	mAb/fusion	VEGF neutralization	Extensive clinical	No	Contraindicated
Faricimab	Bispecific mAb	VEGF + Ang-2	Phase III clinical	No	Contraindicated

## Data Availability

No new data were created or analyzed in this study. Data sharing is not applicable to this article.
